# Exosomes with overexpressed miR 147a suppress angiogenesis and infammatory injury in an experimental model of atopic dermatitis

**DOI:** 10.1038/s41598-023-34418-y

**Published:** 2023-06-01

**Authors:** Chenlong Shi, Sujun Pei, Ying Ding, Congmin Tao, Yuanzheng Zhu, Ying Peng, Wei Li, Yangyan Yi

**Affiliations:** Department of Plastic Surgery, The Second Affiliated Hospital of Nanchang University, No. 1, Minde Road, Nanchang, 330006 Jiangxi People’s Republic of China

**Keywords:** Cell biology, Molecular biology

## Abstract

Atopic dermatitis is defined as an intensely systemic inflammation among skin diseases. Exosomes derived from adipose-derived stem cells may be a novel cell-free therapeutic strategy for atopic dermatitis treatment. This study aims to elucidate the possible underlying mechanism of adipose-derived stem cells-exosomes harboring microRNA-147a in atopic dermatitis pathogenesis. BALB/c mice treated with *Dermatophagoides farinae* extract/2,4-dinitrochlorobenzene were defined as a mouse model of atopic dermatitis, either with inflamed HaCaT cells and HUVECs exposed with TNF-α/IFN-γ stimulation were applied for a cell model of atopic dermatitis. The concentrations of IL-1β and TNF-α in the supernatants were examined by ELISA. Cell viability and migration were assessed by MTT and Transwell assay. The apoptosis was examined using flow cytometry and TUNEL staining. The tube formation assay was employed to analyzed angiogenesis. The molecular regulations among miR-147a, MEF2A, TSLP and VEGFA were confirmed using luciferase reporter assay, either with ChIP. microRNA-147a was markedly downregulated in the serum and skin samples of atopic dermatitis mice, of which overexpression remarkably promoted HaCaT cell proliferation, meanwhile inhibiting inflammatory response and cell apoptosis. microRNA-147a in adipose-derived stem cells was subsequently overexpressed, and exosomes (Exos-miR-147a mimics) were collected. Functionally, exos-microRNA-147a mimics attenuated TNF-α/IFN-γ-induced HaCaT cell inflammatory response and apoptosis, and suppressed HUVECs angiogenesis. Encouraging, molecular interaction experiments revealed that exosomal microRNA-147a suppressed TNF-α/IFN-γ-induced HUVECs angiogenesis by targeting VEGFA, and exosomal microRNA-147a repressed HaCaT cells inflammatory injury through the MEF2A-TSLP axis. Mechanistically, exosomal microRNA-147a repressed pathological angiogenesis and inflammatory injury during atopic dermatitis progression by targeting VEGFA and MEF2A-TSLP axis. microRNA-147a-overexpressing adipose-derived stem cells-derived exosomes suppressed pathological angiogenesis and inflammatory injury in atopic dermatitis by targeting VEGFA and MEF2A-TSLP axis.

## Introduction

Atopic dermatitis (AD) is a type of chronic, inflammatory, and highly sensitive skin disease, which is characterized by the infiltration of immune cells, including mast cells and eosinophils^1^. As a common disease, AD affects 20% of children and 3% of adults worldwide, and the number of AD patients is rapidly increasing^2^. Although the current treatment drugs for AD such as cortisol, calcineurin inhibitors, and immunosuppressants have shown good therapeutic effects on AD, these drugs might be inappropriate for long-term use because of their adverse effects^3^. At present, systemic JAK inhibitors such as Dupilumab and upadacitinib are currently approved or being developed for the treatment of AD^4,5^, while some adverse effects were reported^5^. Dupilumab facial redness is a development of an eczematous facial rash after initiation of dupilumab and is an adverse event not described in clinical trials^6^. And rates of serious infection, eczema herpeticum, herpes zoster, and laboratory-related adverse events were higher for patients who received upadacitinib^5^. Many further monoclonal antibodies are under study in AD, mainly targeting type 2 inflammation^7^. However, the efficacy of these monoclonal antibodies is not ideal. For example, secukinumab, an anti-IL-17A monoclonal antibody, was investigated in AD patients, but there was no significant improvement compared to placebo^8^. In addition, IL-17C antagonist (MOR106) in experimental models reduced skin inflammation^9,10^, but clinical studies (NCT03568071, NCT03864627, NCT03689829) were prematurely stopped for futility. Therefore, it’s urgent to develop effective and safe treatment strategies for AD. The pathogenesis of AD is very complex and is related to many factors, including genetic background, environmental factors, pathological angiogenesis, microvessel functional alterations and abnormal immune response^11^. Angiogenesis, the growth of new blood vessels from pre-existing blood vessels, is associated with inflammation in various pathological conditions. Notably, angiogenesis is associated with the pathogenesis and progression of AD^12^. Vascular endothelial growth factor (VEGF) as well as proinflammatory cytokines are unregulated in AD and induce angiogenesis^12^; therefore, inhibition of angiogenesis might be an effective treatment strategy for AD.

Mesenchymal stem cells (MSCs) are a type of pluripotent stem cells that are widely used in cell therapy^13^. Adipose-derived stem cells (ADSCs) are a subpopulation of cells with multiple differentiation potentials^14^. Many reports have revealed the therapeutic effects of ADSCs on AD^15,16^. As widely illustrated, paracrine activity is the most important mediator for MSCs to achieve their biological roles^17–19^. Exosome is recognized as the key component that regulates MSCs’ paracrine activity^20,21^. Exosomes transport various molecules from MSCs to target cells to mediate biological processes^22^. To date, a few clinical studies have been registered to analyze the therapeutic values of tissue-derived MSCs, engineered MSCs, and MSC-derived exosomes. Encouragingly, favorable clinical outcomes indicating safety and efficacy have been obtained from several trials^23^, revealing the potential of MSCs-derived exosomes as a safe cell-free therapeutic strategy. Notably, it has been widely illustrated that ADSCs-derived exosomes have good therapeutic effects on AD preclinically and clinically. As proof, Park et al*.* demonstrated that topical application of human ADSCs-derived exosomes could remarkably improve dupilumab-related facial redness in patients with severe AD^24^. In addition, as previously described, ADSCs-derived exosome injection could reduce AD pathological symptoms, such as clinical score, serum IgE level, lesional mast cell infiltration as well as CD86^+^ CD206^+^ cell infiltration^25^. Nevertheless, the function of ADSCs-exosomes in regulating angiogenesis during AD progression and the molecular mechanisms involved have not been elucidated.microRNAs (miRNAs) refer to noncoding RNA molecules of about 20 nucleotides in length^26^. miRNAs dysregulation is an important inducement for the pathogenesis and development of AD^27^. As proof, miR-335 expression was markedly reduced in AD lesional skin tissues, and its overexpression rescued AD defective skin barrier^28^. microRNA-147a (miR-147a) is a recent research hotspot in inflammation. For instance, miR-147a upregulation could alleviate lipopolysaccharide (LPS)-induced lung inflammation^29^. Notably, it was also observed that miR-147a overexpression repressed high glucose-induced endothelial cell inflammation and oxidative stress^30^. Considering the essential modulation of miR-147a in the inflammatory response, miR‐147a‐mediated therapy might be a potential therapeutic strategy for AD. However, there are difficulties in delivering miR-147a to AD lesional areas. With the development of cell‐free transplantation strategies, we considered whether ADSCs-derived exosomes could be applied as a carrier of miR-147a to achieve a combination of their functions and effects.

Herein, the function of exosomes derived from miR‐147a‐overexpressing ADSCs in combating AD and the molecular mechanisms involved were investigated. We demonstrated that exosomes derived from miR-147a-overexpression ADSCs suppressed pathological angiogenesis during AD progression by targeting VEGFA and myocyte enhancer factor 2A (MEF2A)-thymic stromal lymphopoietin (TSLP) axis, providing a potential novel therapeutic strategy for the treatment of AD.

## Materials and methods

### Induction of AD-like lesions in the mouse ear

A total of 30 female BALB/c mice (6-week-old) were purchased from SLACOM (Shanghai, China). The AD mice model was induced by *Dermatophagoides farinae* extract (DFE)/2,4-dinitrochlorobenzene (DNCB) treatment as previously described^31^. In brief, DFE, purchased from Greer Laboratories (NC, USA), was dissolved in PBS containing 0.5% Tween 20. And 1% DNCB, obtained from Sigma-Aldrich (MO, USA), was dissolved in an acetone/olive oil (1:3) solution. Mice were randomized into two groups (n = 15/group): the normal group and the AD group. Mice were anesthetized with 1% isoflurane, and surgical tape (Nichiban, Tokyo, Japan) was employed to strip the surfaces of both ear lobes gently. Then, each ear was painted with 20 μl of DFE and 20 μl DNCB repeated once a week for 4 weeks. Ear swelling was detected 24 h after DFE/DNCB treatment by a micrometer (Mitutoyo, Tokyo, Japan). Then, mice were euthanized with carbon dioxide (CO_2_), and the blood samples and ear tissues were collected for further experiments. All procedures involving animals were conducted in accordance with the ARRIVE guidelines and were also carried out strictly according to the guide for the Care and Use of Laboratory Animals, under the approval of the Animal Care and Ethical Committee of The Second Affiliated Hospital of Nanchang University.

### Cell culture and treatment

Human epidermal keratinocyte cells (HaCaT cells), human umbilical vein endothelial cells (HUVECs) and ADSCs were obtained from the Chinese Academy of Sciences (Shanghai, China). HUVECs and ADSCs were cultured in RPMI 1640 (Gibco, MD, USA) mixed with 10% FBS (Gibco) at 37 °C with 5% CO_2_. HUVECs were cultured in endothelial cell medium (ECM; ScienCell, CA, USA) with 10% FBS (Gibco), 1% endothelial cell growth supplement (ScienCell), and 1% penicillin and streptomycin (ScienCell) at 37 °C with 5% CO_2_. HaCaT cells and HUVECs were subjected to 10 ng/ml recombinant tumor necrosis factor (TNF)-α and 10 ng/ml interferon (IFN)-γ (R&D systems, MN, USA) for 6 h. For exosomes treatment, cells were subjected to exosomes (30 µg/mL) for 24 h.

### Cell transfection

The overexpression plasmid of MEF2A (OE-MEF2A), the short hairpin RNA of MEF2A (sh-MEF2A), mimic/inhibitor of miR-147a and their negative controls were purchased from GenePharma (Shanghai, China). Before transfection, 1 × 10^6^ cells were cultured in 6‐well plates with 2 mL complete medium for 24 h until they were 90% confluent. Then cells were transfected with 100 ng plasmids and 200 nmol/L miR‐147a mimics, miR‐147a inhibitors or their negative control by using Lipofectamine™ 3000 (Invitrogen, CA, USA) for 48 h following the instructions of the manufacturer.

### Cell counting kit-8 (CCK-8) assay

HT22 cells were cultured in 24-well plates (2 × 10^4^ cells/well) for 24 h and incubated with CCK-8 solution (10 μL, Sangon, Shanghai, China) at 37 °C for 3 h. Absorbance at 450 nm was subsequently analyzed by using a spectrophotometer (Thermo Scientific, MA, USA).

### TdT-mediated dUTP nick-end labeling (TUNEL) staining

Cells were fixed and permeabilized. TUNEL staining was performed with the kit obtained from Roche (Basel, Switzerland). The nucleus was stained with DAPI (Sangon, Shanghai, China). Cells were observed with a fluorescence microscope (Olympus, Tokyo, Japan).

### Enzyme-linked immunosorbent assay (ELISA)

The levels of TNF-α and IL-1β were examined by the human TNF-α ELISA kit (Beyotime, Shanghai, China, PT518) and the human IL-1β ELISA kit (Beyotime, PI305) according to the manual. The data were analyzed in the microplate reader (Bioteke, Beijing, China).

### Flow cytometry

Cells were re-suspended in 500 μL of binding buffer (Beyotime) and then incubated with 10 μL Annexin V-FITC and 5 μL PI stain for 10 min. Samples were immediately analyzed using flow cytometry (Becton, Dickinson and Company, NJ, USA).

### Isolation and identification of exosomes

Exosomes were isolated with ExoQuick-TC (System Bioscience, CA, USA) according to the manual. The markers (CD63, Calnexin and TSG101) were verified by western blot. The exosomes were subjected to nanoparticle tracking analysis (Malvern Panalytical, Malvern, UK) to detect size.

### Transmission electron microscopy (TEM)

Exosomes were loaded and incubated with phosphotungstic acid (Sigma-Aldrich, MO, USA) for 1 min followed by examination using TEM (HITACHI, Tokyo, Japan).

### Exosome labeling and uptake

Exosomes were resuspended in 1 mL Diluent C and incubated with 4 µL PKH26 dye (Sigma-Aldrich) for 4 min. Then MLE-12 cells were subjected to labeled exosomes for 12 h. Then cells were fixed, stained with DAPI and visualized with a fluorescence microscope (Olympus).

### Dual-luciferase reporter gene assay

TSLP promoter fragment was amplified by PCR. Site-directed mutagenesis was performed using a site mutation kit (Stratagene, CA, USA). Wild type (WT) and mutant (MUT) of TSLP promoter sequences were cloned into the pmiRGLO vector (Promega, WI, USA) Then, cells were co-transfected with TSLP promoter-WT or TSLP promoter-MUT and sh-NC/OE-NC or sh-MEF2A/OE-MEF2A. The luciferase activity was subsequently assessed. The same method was employed to analyze the interaction between miR-147a and MEF2A/VEGFA.

### Chromatin immunoprecipitation (ChIP) assay

Cells were fixed, quenched and sonicated. The cell lysate was subsequently incubated with anti-MEF2A (Abcam, 1:100, ab76063) or anti-IgG (Abcam, 1:100, ab172730) at 4 °C overnight. The enriched DNA was subjected to RT-qPCR analysis.

### Transwell migration assay

DMEM containing 1 × 10^4^ cells (500 μL) were placed in the upper chamber (Becton, Dickinson and Company) and complete DMEM (1000 μL) was placed in the bottom chamber. The cells were then fixed and stained with hematoxylin for 30 min after 12 h. Cells on the upper side of the filter were removed. Cells were photographed with a microscope (Olympus).

### Tube formation assay

HUVECs were seeded into 96-well plates precoated with Matrigel (1:1 ratio) (Becton, Dickinson and Company). After cell attachment, cells were cultured in DMEM for 24 h. Cells were photographed with a microscope (Olympus).

### Real time quantitative polymerase chain reaction (RT-qPCR)

Total RNA was extracted with TRIzol (ThermoFisher Scientific, MA, USA). The cDNA was synthesized by the HiFiScript cDNA synthesis kit (Toyobo, Tokyo, Japan) and the first-strand cDNA synthesis kit (Sangon), and subjected to RT-qPCR assay with SYBR (ThermoFisher Scientific). GAPDH and U6 were employed as the reference genes. The data were analyzed with 2^−ΔΔCT^ method. The primers were listed as follows (5’-3’):

- miR-147a (F): GCGGGCGTGTGTGAAATGC
- miR-147a (R): ATCCAGTGCAGGGTCCGAGG
- IL-1β (F): AAAGCTTGGT GATGTCTGGTC
- IL-1β (R): GGACATGGAGAACACCACTTG
- TNF-α (F): CTCAGCAAGGACAGCAGAGG
- TNF-α (R): ATGTGGCGTCTGAGGGTTGTT
- VEGFA (F): AGGGCAGAATCATCACGAAGT
- VEGFA (R): AGGGTCTCGATTGGATGGCA
- TLSP (F): ATGTTCGCCATGAAAACTAAGGC
- TLSP (R): GCGACGCCACAATCCTTGTA
- MEF2A (F): GGTCTGCCACCTCAGAACTTT
- MEF2A (R): CCCTGGGTTAGTGTAGGACAA
- GAPDH (F): ATGACTCTACCCACGGCAAG
- GAPDH (R): GGAAGATGGTGATGGGTTTC
- U6 (F): CTCGCTTCGGCAGCACA
- U6 (R): AACGCTTCACGAATTTGCGT.

### Western blot

The proteins were isolated with RIPA, which were further transferred to a PVDF membrane (Millipore, MA, USA). Then, membranes were incubated overnight with antibodies including anti-MEF2A (Abcam, 1:1000, ab76063), anti-VEGFA (Abcam, 1:1000, ab46154) and anti-GAPDH (Abcam, 1:10,000, ab8245). After washing with PBS-T, membranes were then incubated with the secondary antibody (Abcam, 1:10,000, ab7090) for 60 min. The membranes were visualized and imaged by a GEL imaging system (Bio-Rad, CA, USA).

### Data analysis

Statistical data were analyzed by SPSS 19.0 (IBM, Armonk, NY) and expressed as means ± S). Between-group differences and multi-group comparisons were determined using Student’s t-test and one-way ANOVA, respectively. *p* < 0.05 was considered to represent a significant difference. All the tests conducted in this work were repeated at least three times.

### Ethical approval and consent to participate

All procedures involving animals were carried out strictly according to the guide for the Care and Use of Laboratory Animals, under the approval by the Animal Care and Ethical Committee of The Second Affiliated Hospital of Nanchang University.

## Results

### Expression pattern of miR-147a in serum and skin tissues of AD mice

**Figure 1:**
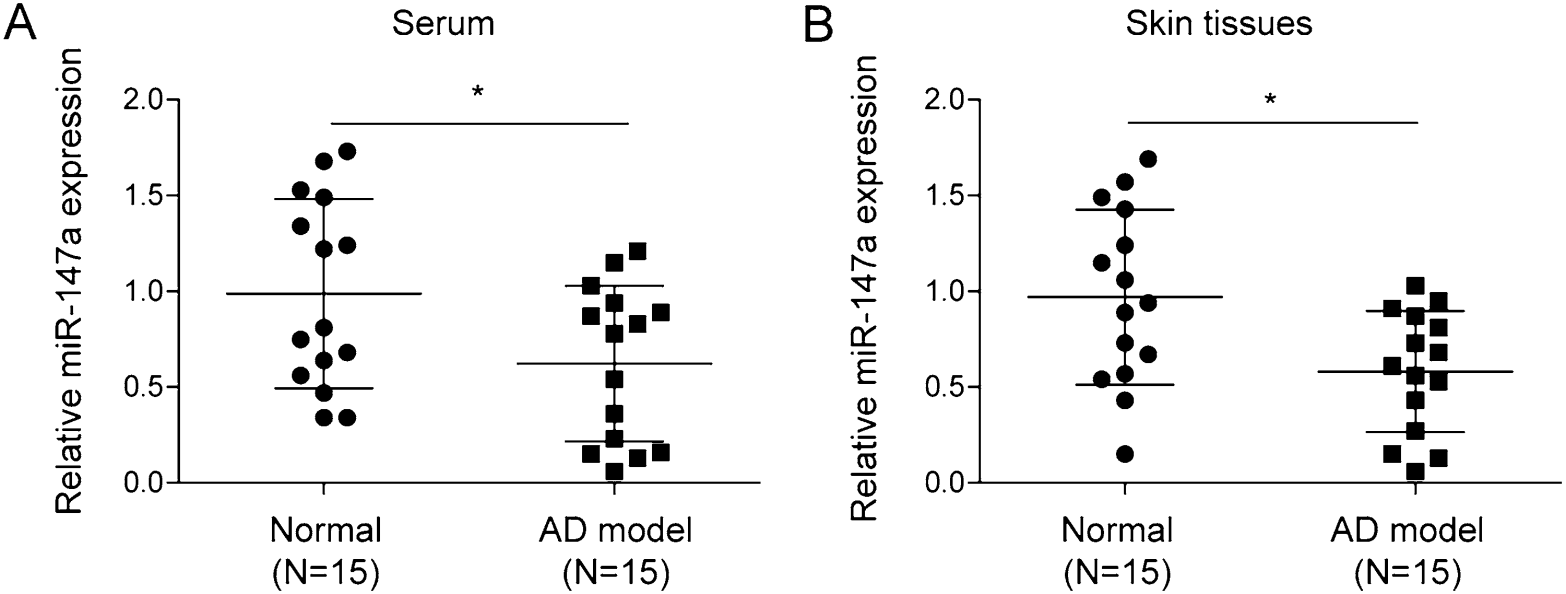
Expression pattern of miR-147a in serum and skin tissues of AD mice. miR-147a expression in serum and skin samples from AD mice and normal mice was assessed by RT-qPCR. Data were expressed as mean ± SD. n = 15. *p < 0.05, **p < 0.01, ***p < 0.001.

As demonstrated in Fig. 1A,B, miR-147a expression was significantly reduced in serum and lesions from AD mice compared to that in serum and skin tissues from the normal mice. It was concluded that miR-147a was downregulated in AD.

### Reinforced miR-147a attenuated TNF-α/IFN-γ-induced HaCaT cell inflammatory response and cell apoptosis

**Figure 2:**
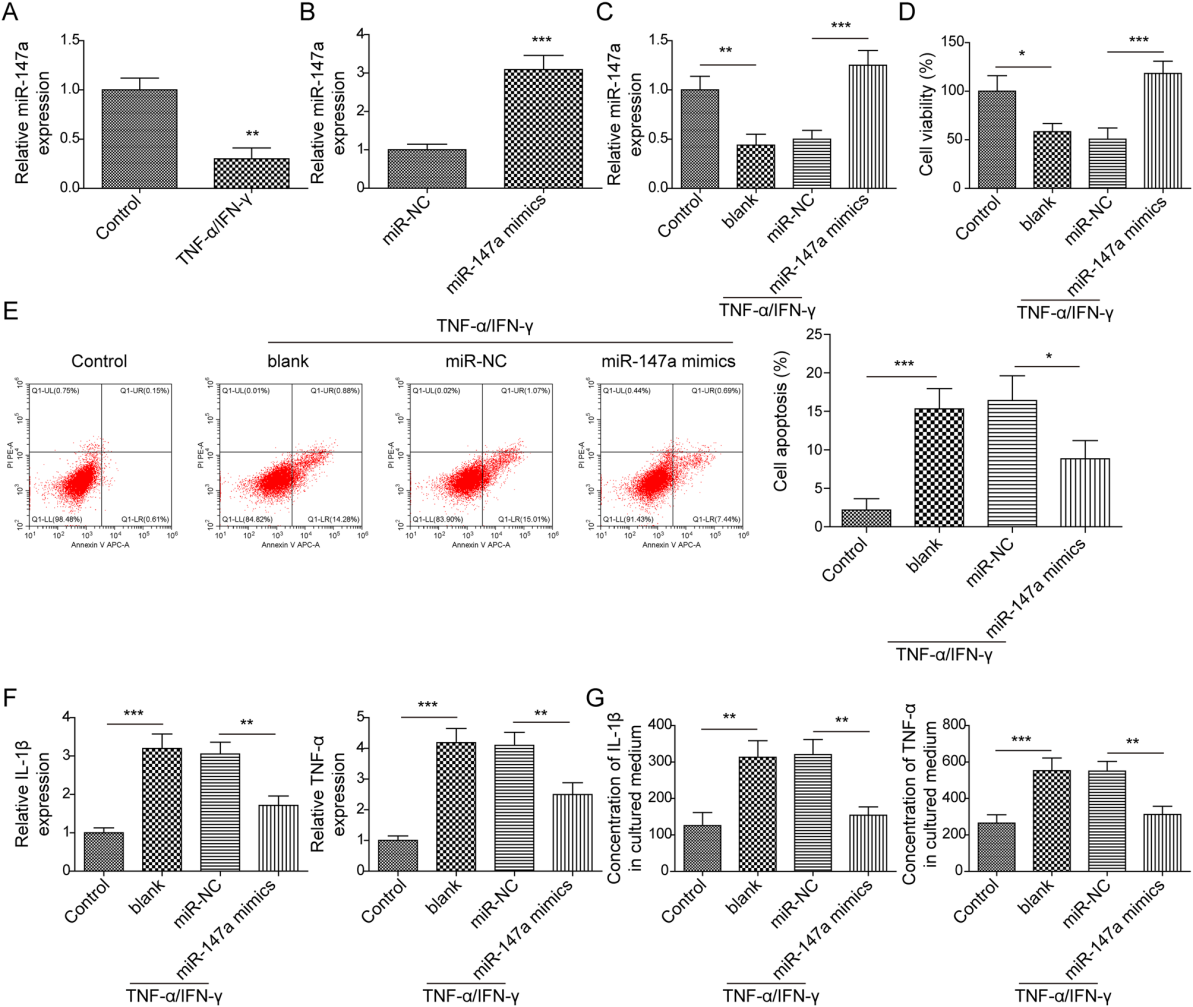
Reinforced miR-147a attenuated TNF-α/IFN-γ-induced HaCaT cell inflammatory response and cell apoptosis. (**A**) miR-147a expression in HaCaT cells after TNF-α/IFN-γ stimulation was determined by RT-qPCR. (**B**) RT-qPCR was employed to detect miR-147a expression in HaCaT cells following miR-147a mimics or mimics NC transfection. miR-147a was induced in HaCaT cells following TNF-α and IFN-γ stimulation for 24 h. (**C**) miR-147a expression in HaCaT cells was examined by RT-qPCR. (**D**) CCK8 assay was performed to analyze HaCaT cell viability. (**E**) Cell apoptosis was determined by flow cytometry. (**F**, **G**) IL-1β and TNF-α levels were detected by RT-qPCR and ELISA. Data were expressed as mean ± SD. All our data were obtained from three independent experiments. *p < 0.05, **p < 0.01, ***p < 0.001.

As reported, keratinocytes from AD patients secrete high concentrations of inflammatory cytokines following TNF-α/IFN-γ stimulation^32^. Therefore, TNF-α/IFN-γ-treated HaCaT cells were employed to probe the role of miR-147a in AD in vitro. It was observed that miR-147a in HaCaT cells was markedly reduced following TNF-α/IFN-γ stimulation (Fig. 2A). We subsequently induced miR-147a overexpression in HaCaT cells by transfecting miR-147a mimics into cells (Fig. 2B). As revealed in Fig. 2C, miR-147a overexpression reversed the inhibitory effect of TNF-α/IFN-γ on miR-147a expression in HaCaT cells. The results of CCK-8 assay subsequently revealed that HaCaT cell vitality was markedly suppressed by TNF-α/IFN-γ stimulation, while this change was reversed by miR-147a overexpression (Fig. 2D). In addition, TNF-α/IFN-γ treatment induced HaCaT cell apoptosis, which was abrogated by miR-147a overexpression (Fig. 2E). Moreover, it was observed that TNF-α/IFN-γ stimulation resulted in increased levels of inflammatory cytokines (IL-1β and TNF-α), which were all abolished by miR-147a overexpression. (Fig. 2F,G). Collectively, miR-147a overexpression remarkably promoted inflamed HaCaT cell proliferation, meanwhile inhibiting inflammatory injury and cell apoptosis.

### Correlation of miR-147a among TLSP and VEGFA expression levels in serum and skin tissues of AD mice

**Figure 3:**
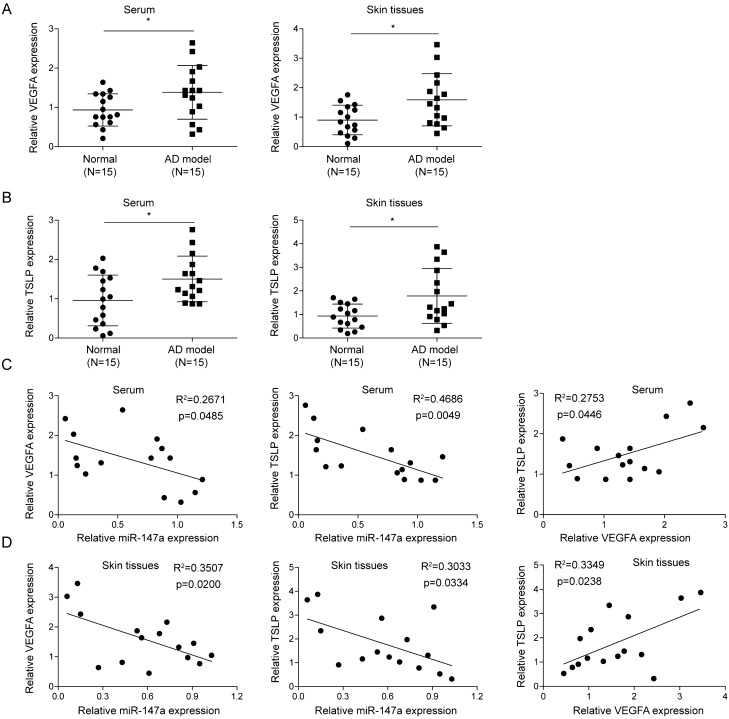
Correlation of miR-147a among TLSP and VEGFA expression levels in serum and skin tissues of AD mice. (**A**, **B**) VEGFA and TSLP expressions in serum and skin samples from AD mice and normal mice were assessed by RT-qPCR. (**C**, **D**) Expression correlations of miR-147a, VEGFA and TSLP in serum and skin tissues of AD mice were analyzed by Spearman correlation analysis. Data were expressed as mean ± SD. n = 15. *p < 0.05, **p < 0.01, ***p < 0.001.

Angiogenesis and changes in microvascular morphology and function are important features of AD^11^. TSLP, an epithelial cell-derived cytokine, is involved in AD pathogenesis, which is also closely related to the angiogenic phenotype^33^. Herein, our results illustrated that TSLP and VEGFA (a key regulator of angiogenesis) expressions were significantly increased in serum and lesions from AD mice compared to those in serum and skin tissues from the normal mice (Fig. 3A,B). It was subsequently revealed that miR-147a expression in AD mice was significantly negatively correlated with VEGFA expression, as well as TSLP expression both in the samples of serum and skin tissues (Fig. 3C,D). Collectively, miR-147a expression was negatively correlated with the expressions of VEGFA and TSLP in AD mice.

### Identification of ADSCs and ADSCs-derived exosomes

**Figure 4:**
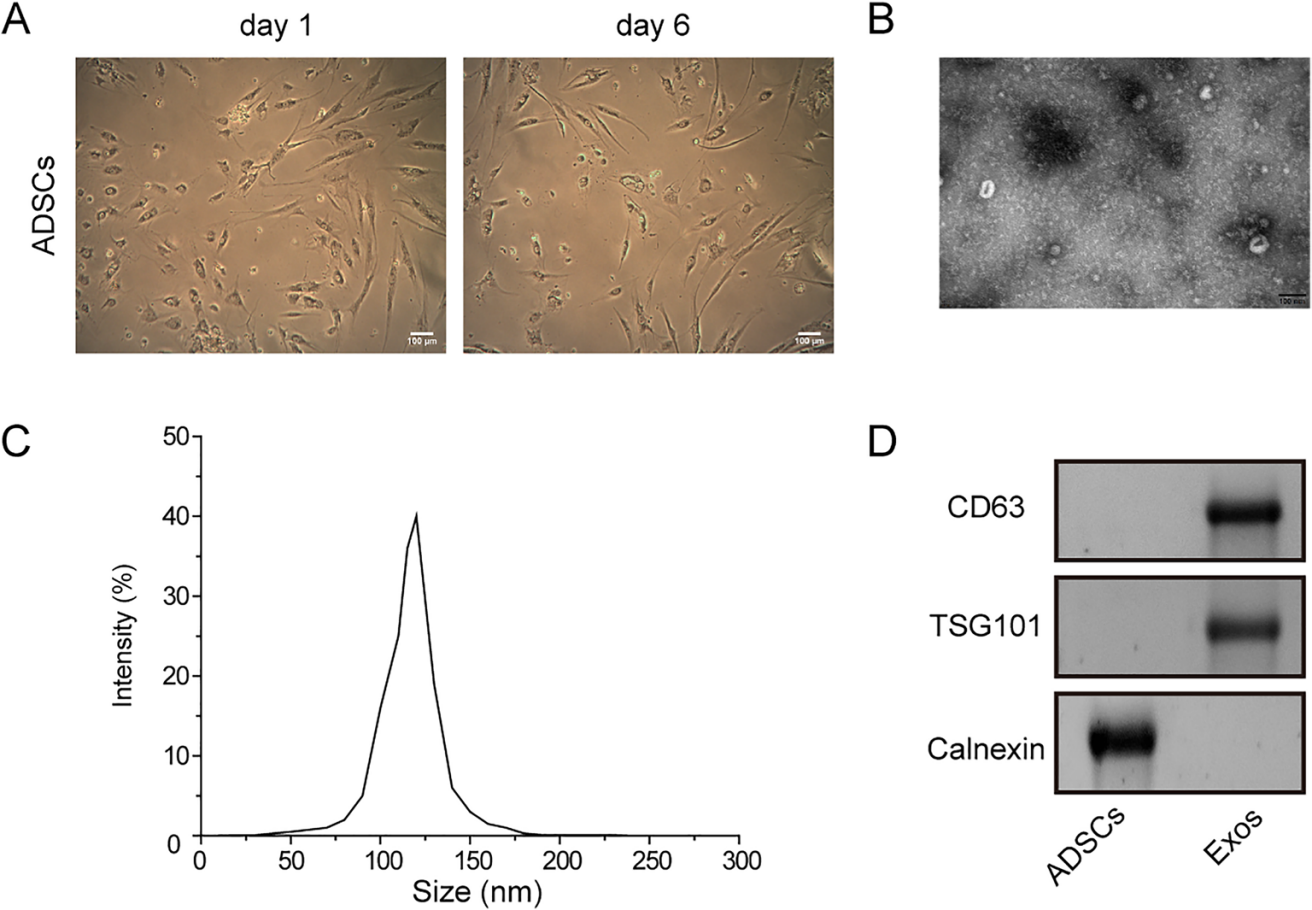
Identification of ADSCs and ADSCs-derived exosomes. (**A**) ADSCs morphology was determined using a microscope. (**B**) Exosome ultrastructure was detected by TEM. (**C**) NTA was used to measure the size distribution of exosomes. (**D**) CD63, TSG101 and calnexin levels were elevated using western blot. Data were expressed as mean ± SD. All our data were obtained from three independent experiments. *p < 0.05, **p < 0.01, ***p < 0.001.

Based on previous studies confirming the therapeutic effect of ADSCs-derived exosomes on AD mouse models and the ability of exosomes to transport miRNAs, we planned to overexpress miR-147a in ADSCs, then isolate exosomes and investigate its therapeutic effect on AD. It was first observed that ADSCs had characteristic morphological features, which exhibited the shape of long spindle under the microscope (Fig. 4A). As displayed in Fig. 4B,C, exosomes had characteristic morphological features under TEM with a size of 50–150 nm. The data of western blot also confirmed the expression of CD63 and TSG101 was positively expressed, while calnexin was absent in exosomes (Fig. 4D). Taken together, we successfully isolated ADSCs and ADSCs-derived exosomes.

### Exosomes from miR-147a-overexpressing ADSCs weakened TNF-α/IFN-γ-mediated impairments on HaCaT cells and HUVECs

**Figure 5:**
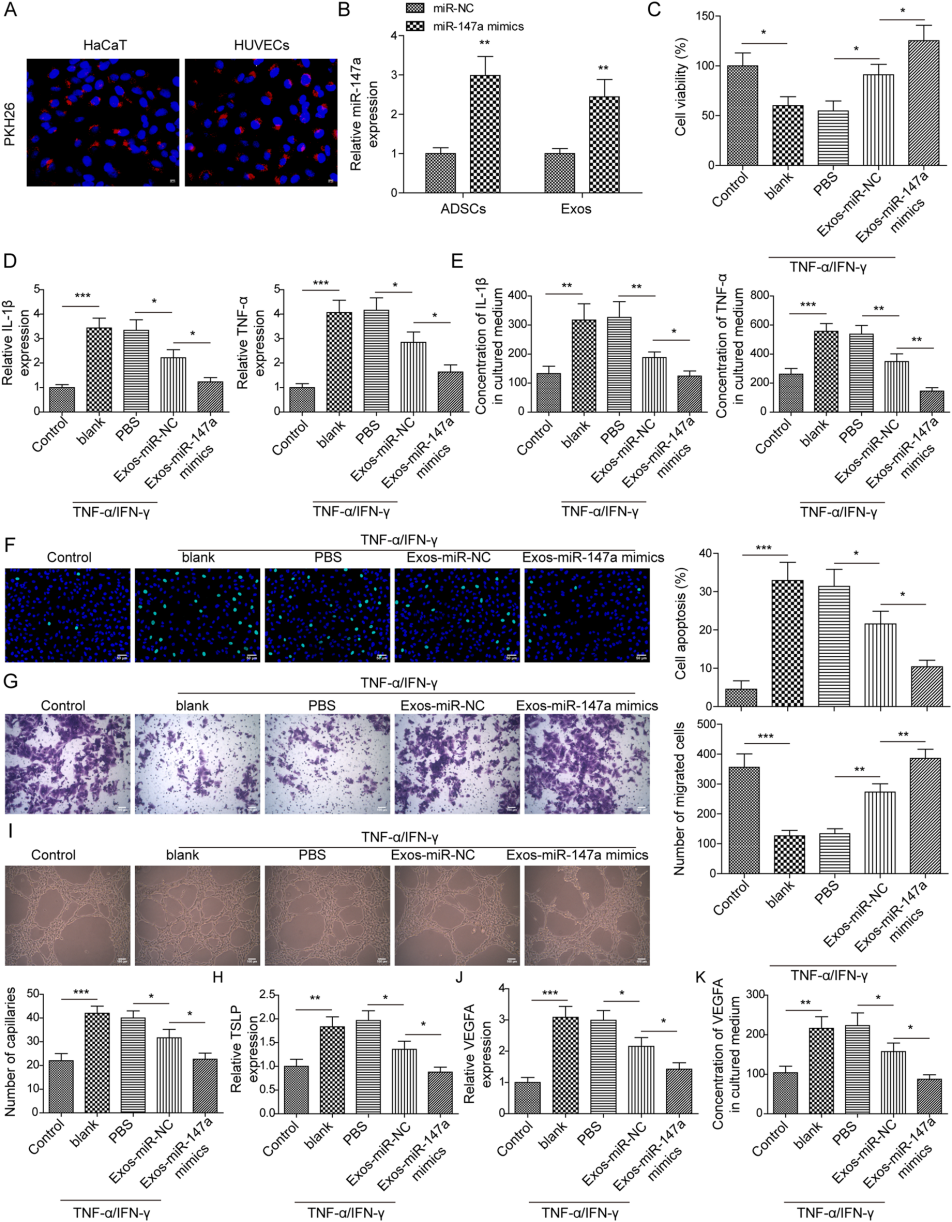
Exosomes from ADSCs-overexpressing miR-147a weakened TNF-α/IFN-γ-mediated impairments on HaCaT cells and HUVECs. (**A**) IF was employed to analyze the uptake of PKH26‐labelled exosomes by HaCaT cells and HUVECs. (**B**) miR-147a overexpression was induced in ADSCs, and miR-147a expression in exosomes and ADSCs was assessed using RT-qPCR. TNF-α/IFN-γ-treated HaCaT cells were subjected to Exos-miR-147a mimics or Exos-miR-NC. (**C**) HaCaT cell viability was examined by CCK8 assay. (**D**, **E**) IL-1β and TNF-α levels in HaCaT cells were detected by RT-qPCR and ELISA. (**F**) HaCaT cell apoptosis was analyzed using TUNEL staining. (**G**) Transwell assay was employed to assess HaCaT cell migration. (**H**) RT-qPCR was employed to examine TSLP expression in HaCaT cells. TNF-α/IFN-γ-treated HUVECs were subjected to Exos-miR-147a mimics or Exos-miR-NC. (**I**) HUVEC angiogenesis was analyzed by tube formation assay. (J-K) VEGFA level in HUVECs was detected by RT-qPCR and ELISA. Data were expressed as mean ± SD. All our data were obtained from three independent experiments. *p < 0.05, **p < 0.01, ***p < 0.001.

As demonstrated in Fig. 5A, after incubation with HaCaT cells and HUVECs, PKH26‐labelled exosomes presented red fluorescence in cells, indicating exosomes could be taken up by cells. To explore whether the exosomes from miR-147a-overexpressing ADSCs could be adopted as a potential treatment for AD, miR-147a in ADSCs was overexpressed, and exosomes (Exos-miR-147a mimics) were collected for subsequent experiments. It turned out that miR-147a expression was significantly higher in miR-147a mimics-transfected ADSCs and their exosomes (Exos-miR-147a mimics) than that in mimics NC-transfected ADSCs and their exosomes (Exos-miR-NC) (Fig. 5B), suggesting that ADSCs efficiently packaged miR-147a into secreted exosomes. The result of CCK8 assay illustrated that Exos-miR-NC treatment abrogated TNF-α/IFN-γ stimulation’s repression on HaCaT cell viability, and it was also observed that Exos-miR-147a mimics-treated HaCaT cell viability was increased relative to Exos-miR-NC-treated cells (Fig. 5C). The promoting of TNF-α/IFN-γ stimulation on IL-1β and TNF-α levels in HaCaT cells was abolished by Exos-miR-NC treatment, and the results also revealed a decrease of IL-1β and TNF-α levels in Exos-miR-147a mimics-treated HaCaT cells relative to the levels in Exos-miR-NC-treated cells (Fig. 5D,E). In addition, Exos-miR-NC treatment eliminated TNF-α/IFN-γ stimulation’s facilitation on HaCaT cell apoptosis, and this effect was further enhanced by miR-147a mimics pretreatment (Fig. 5F). The result of Transwell assay displayed that the inhibitory effect of TNF-α/IFN-γ stimulation on HaCaT cell migration was reversed by Exos-miR-NC treatment, and it demonstrated that an increase of migrative cells among Exos-miR-147a mimics-treated HaCaT cells (Fig. 5G). Moreover, TSLP expression in HaCaT cells was markedly upregulated by TNF-α/IFN-γ stimulation, whereas this change was abolished by Exos-miR-NC treatment (Fig. 5H). It was also observed that TSLP expression was reduced in Exos-miR-147a mimics group compared to that in Exos-miR-NC group (Fig. 5H). The effect of Exos-miR-147a mimics on angiogenesis during AD progression was also analyzed, and the result of tube formation assay revealed that Exos-miR-NC treatment reversed the promoting effect of TNF-α/IFN-γ stimulation on the angiogenesis of HUVECs, and it was also found that Exos-miR-147a mimics-treated HUVEC angiogenesis was reduced relative to Exos-miR-NC-treated cells (Fig. 5I). Meanwhile, the promoting of TNF-α/IFN-γ stimulation on the mRNA and secretion levels VEGFA in HUVECs was abolished by Exos-miR-NC treatment, and the results also revealed a decrease of VEGFA level in Exos-miR-147a mimics-treated HUVECs relative to the levels in Exos-miR-NC-treated cells (Fig. 5J,K). In conclusion, exosomes from miR-147a-overexpressing ADSCs could attenuate TNF-α/IFN-γ-induced HaCaT cell inflammatory response and cell apoptosis, and suppress HUVEC angiogenesis.

### MEF2A was identified to be a transcriptional activator of TLSP

**Figure 6:**
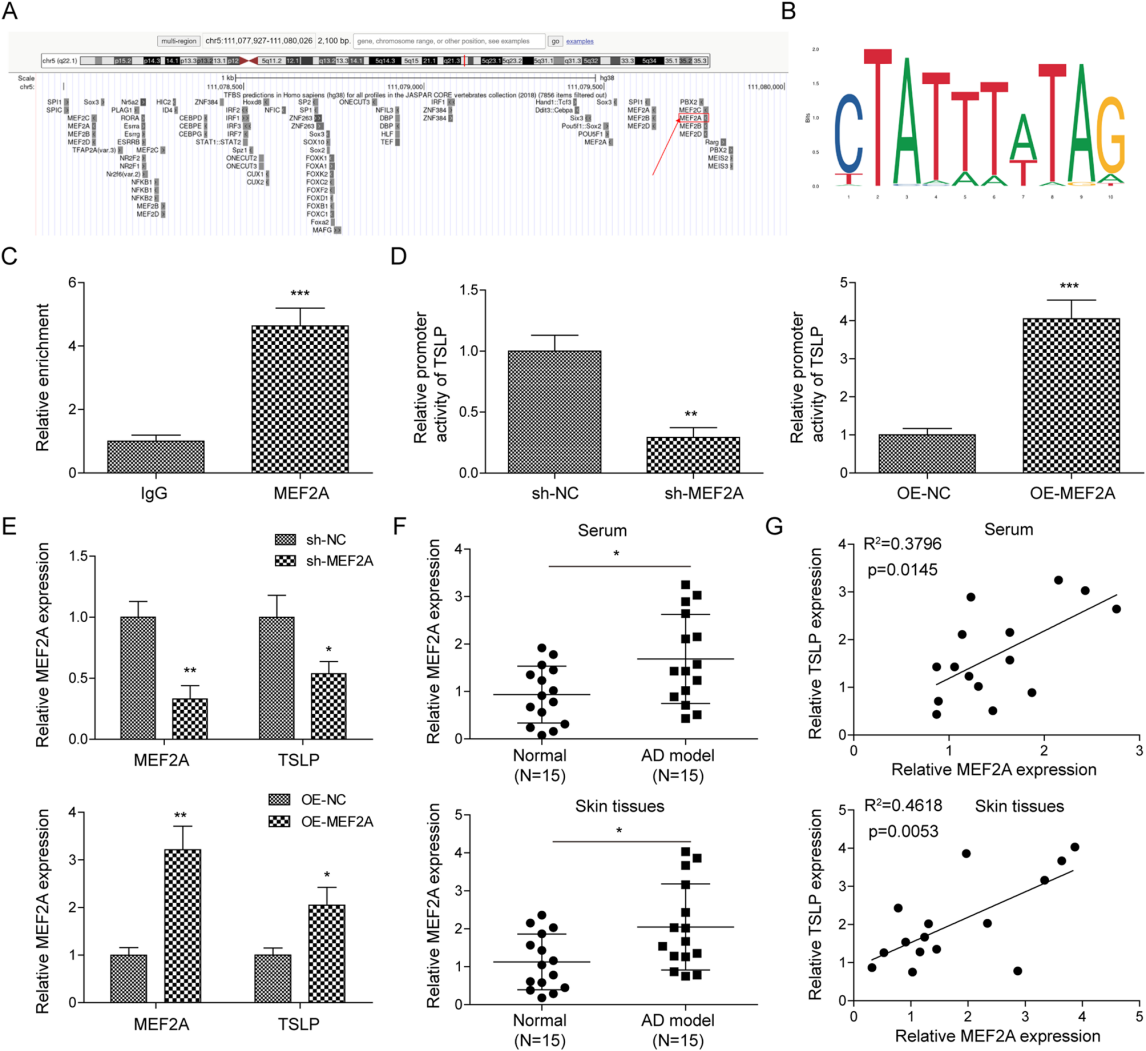
MEF2A was identified to be a transcriptional activator of TLSP. (**A**, **B**) UCSC and JASPAR websites were employed to predict the binding site between TSLP promoter and MEF2A. (**C**, **D**) ChIP and dual-luciferase reporter gene assays were employed to analyze the interaction between MEF2A and the TSLP promoter. (**E**) MEF2A and TSLP mRNA levels in HaCaT cells after MEF2A knockdown/overexpression were assessed using RT-qPCR. (**F**) MEF2A mRNA level in serum and skin samples from AD mice and normal mice was assessed by RT-qPCR (n = 15). (**G**) Expression correlation of MEF2A and TSLP in serum and skin tissues of AD mice was analyzed by Spearman correlation analysis. Data were expressed as mean ± SD. All our data were obtained from three independent experiments. *p < 0.05, **p < 0.01, ***p < 0.001.

Subsequently, we further explored the downstream molecular mechanisms by which miR-147a regulated AD progression. Based on bioinformatics prediction, we did not find a potential complementary binding site between miR-147a and the 3'-UTR region of TSLP. Therefore, we speculated that miR-147a might affect TSLP expression by regulating the upstream transcription factor of TSLP. By using UCSC (Fig. 6A) and JASPAR (Fig. 6B) websites, it was predicted TSLP promoter had a potential binding site to transcription factor MEF2A. ChIP assay further revealed that MEF2A directly bound with the TSLP promoter (Fig. 6C). Meanwhile, by using dual-luciferase reporter gene assay, it was found that MEF2A transcriptionally activated TSLP expression (Fig. 6D). In addition, it was observed that TSLP expression was markedly suppressed/promoted by MEF2Aknockdown/overexpression (Fig. 6E). MEF2A expression in serum and skin tissues of AD mice was also examined, the results illustrated that MEF2A was significantly upregulated in serum and lesions from AD mice compared to that in serum and skin tissues from the normal mice (Fig. 6F). Spearman correlation analysis subsequently revealed that MEF2A expression in serum and skin tissues of AD mice was significantly positively correlated with TSLP expression (Fig. 6G). In summary, MEF2A functioned as a transcriptional activator of TLSP during AD progression.

### miR-147a negatively regulated expression levels of MEF2A and VEGFA via a direct binding relationship

**Figure 7:**
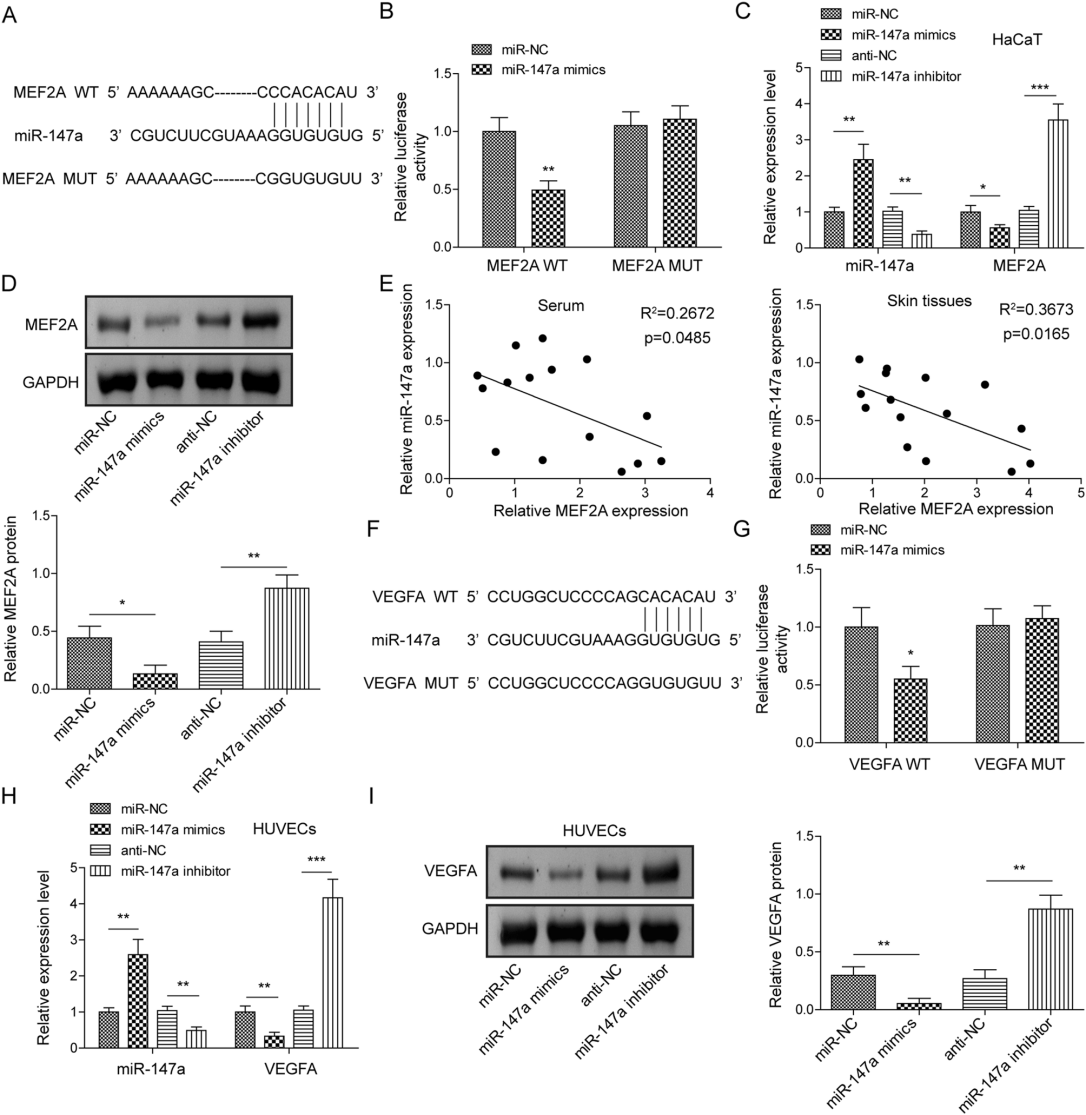
miR-147a negatively regulated expression levels of MEF2A and VEGFA via direct a binding relationship. (**A**) Starbase was employed to predict the complementary binding site between miR-147a and MEF2A. (**B**) Dual-luciferase reporter gene assay was carried out to analyze the interaction between miR-147a and MEF2A. (**C**) miR-147a and MEF2A expressions in HaCaT cells following miR-147a mimics/inhibitor transfection were assessed using RT-qPCR. (**D**) The protein level of MEF2A in HaCaT cells following miR-147a mimics/inhibitor transfection was assessed using western blot. (**E**) The expression correlation of miR-147a and MEF2A in the serum and skin tissues of AD mice was analyzed by Spearman correlation analysis. (**F**) Starbase was employed to predict the complementary binding site between miR-147a and VEGFA. (**G**) Dual-luciferase reporter gene assay was carried out to analyze the interaction between miR-147a and VEGFA. (**H**) miR-147a and VEGFA expressions in HUVECs following miR-147a mimics/inhibitor transfection were assessed using RT-qPCR. (**I**) VEGFA protein level in HUVECs following miR-147a mimics/inhibitor transfection was assessed using western blot. Data were expressed as mean ± SD. All our data were obtained from three independent experiments. *p < 0.05, **p < 0.01, ***p < 0.001.

We subsequently aimed to probe the interaction between MEF2A/VEGFA in regulating AD progression. As illustrated in Fig. 7A, Starbase bioinformatics prediction revealed a complementary binding site between miR-147a and MEF2A. Notably, luciferase reporter assay confirmed that miR-147a mimics also significantly decreased the luciferase activity of MEF2A-WT, while it did not act on MEF2A-MUT activity (Fig. 7B). In addition, it was observed that MEF2A expression level in HaCaT cells was markedly suppressed/increased by miR-147a mimics/inhibitor transfection (Fig. 7C,D). Moreover, miR-137a expression in serum and skin tissues of AD mice was significantly negatively correlated with MEF2A expression (Fig. 7E). Meanwhile, by using Starbase, miR-147a was predicted to show the complementary binding sites of VEGFA (Fig. 7F). And the direct binding relationship among miR-147a and VEGFA was confirmed using luciferase reporter assay, which presented that miR-147a mimics dramatically decreased the luciferase activity of VEGFA-WT, rather than VEGFA-MUT (Fig. 7G). And, as expected, miR-147a overexpression/inhibition resulted in reduced/increased VEGFA expression level in HUVECs (Fig. 7H,I). It was concluded that miR-147a suppressed MEF2A expression in HaCaT cells and VEGFA expression in HEVECs through a direct binding relationship.

## Discussion

AD is an incurable relapsing inflammatory skin disease. The current treatment drugs for AD such as cortisol, and immunosuppressants have shown good therapeutic effects on AD, while these drugs might be inappropriate for long-term use because of their adverse effects^3^. The potential use of MSCs has been a hot topic in tissue repair research^34^. ADSCs are characterized by pluripotent differentiation and are extremely easy to obtain^35^. Recent studies have revealed that the allergic progress in AD could be inhibited by ADSCs^36,37^. Exosomes secreted by MSCs are the main mediators for MSCs’ paracrine activity^38^. As previously reported, as a cell-free therapy, ADSCs-derived exosomes could reduce AD pathological symptoms^25^. Interestingly, MSCs-secreted exosomes are considered to be important mediators of cellular communication by transferring multiple molecules to recipient cells^39^. According to the potential therapeutic effect of exosomes for AD and their carrier potential, we innovatively developed a novel approach to alleviate AD by overexpressing miR-147a in ADSCs to deliver miR-147a specifically to AD lesioned skin tissue via ADSCs-derived exosomes. Mechanistically, Exosomes derived from miR-147a-overexpressing ADSCs-derived exosomes extenuated pathological angiogenesis and inflammatory response during AD progression by targeting VEGFA and MEF2A-TSLP axis.miRNA-147a dysregulation is related to inflammatory infiltration under pathological conditions. For instance, miR-147a overexpression remarkably repressed high glucose-induced mesangial cell oxidative stress and inflammation^40^. Additionally, miR-147a upregulation alleviated LPS-induced lung inflammation^29^. It’s suggested that miR-147 is an anti-inflammatory miRNA, while it has not been confirmed whether miR-147a is involved in AD progression. Herein, it was observed that miR-147a expression was significantly reduced in serum and lesions from AD mice compared with that of control. In addition, miR-147a was lowly expressed in TNF-α/IFN-γ-treated HaCaT cells, which was a commonly used inflamed keratinocyte model^32^. Further experiments revealed that miR-147a upregulation promoted TNF-α/IFN-γ-treated keratinocyte proliferation and repressed inflammatory injury, suggesting that miR-147a upregulation might be a potential therapeutic strategy for AD. A previous study described that human MSCs-derived extracellular vesicles attenuated macrophage activation by miR-147^41^. Therefore, in this study, we designed a study using miR-147a-overexpressing ADSCs-derived exosomes to deliver miR-147a to AD lesions to inhibit AD progression. We first found that miR-147a expression was significantly higher in miR-147a mimics-transfected AMSCs and their exosomes (Exos-miR-147a mimics), and exosomes could be taken up by HaCaT cells and HUVECs, revealing the ability of exosomes to transport miR-147a. As expected, ADSCs-derived exosomes could promote TNF-α/IFN-γ-treated keratinocyte proliferation and repressed inflammatory injury, and these effects became more pronounced following miR-147a overexpression pretreatment. All these results suggested that treatment of exosomes from miR-147a-overexpressing ADSCs could effectively suppress AD progression in vitro.

Subsequently, we further explored the downstream molecular mechanisms by which miR-147a regulated AD progression. As well known, angiogenesis is associated with inflammation in various pathological conditions. Angiogenesis as well as morphological and functional changes in microvessels are hallmark features of AD^11^. VEGFs are key regulators of angiogenesis, which consist of VEGF-A\B\C\D and placental growth factor. VEGFA is the most important pro-angiogenic factor. It has been described that the level of VEGFA was significantly increased in the serum and skin tissues of AD patients, and its level is correlated with disease activity index^42,43^. Consistently, in the present study, our results revealed that VEGFA was obviously increased in serum and lesions from AD mice, of which expression was also negatively correlated with miR-147a. Interestingly, our mechanistic research identified that miR-147a negatively regulated the expression of VEGFA by directly binding with its 3'-UTR. Functional experiments further demonstrated that exosomes from miR-147a-overexpressing ADSCs could attenuate TNF-α/IFN-γ-induced HUVEC angiogenesis by reducing VEGFA expression. Therefore, it was concluded that exosomal miR-147a suppressed TNF-α/IFN-γ-induced HUVEC pathological angiogenesis by targeting VEGFA (Supplementary File 1).

TSLP signaling plays a key role in AD-like inflammation^44^. TSLP, an epithelial cell-derived cytokine, its genetic polymorphism is associated with persistent AD^45^. As reported, TSLP is involved in Th2 immune responses to promote the pathogenesis of AD^46^. In the current research, it revealed an increasing level of TSLP both in serum and lesions from AD mice, which exerted similar data, for its negative correlation with miR-147a in AD mice by using Spearman correlation analysis. Nevertheless, we did not find a potential complementary binding site between miR-147a and the 3'-UTR region of TSLP by using bioinformatics prediction. Therefore, we speculated that miR-147a might affect TSLP expression by regulating the upstream transcription factor of TSLP. By using UCSC and JASPAR, it was predicted TSLP promoter had a potential binding site to transcription factor MEF2A. MEF2A is reported to function as the upstream transcription factor of many inflammation-related genes^47^. Herein, our results illustrated that MEF2A was markedly upregulated in AD mice, and its expression was significantly positively correlated with TSLP expression. In addition, molecular interaction experiments confirmed that MEF2A functioned as a transcriptional activator of TLSP. Encouragingly, miR-147a negatively regulated MEF2A expression in HaCaT cells via a direct binding relationship. In conclusion, exosomal miR-147a suppressed TNF-α/IFN-γ-induced HaCaT cell inflammatory injury by targeting the MEF2A-TSLP axis.

**Figure 8:**
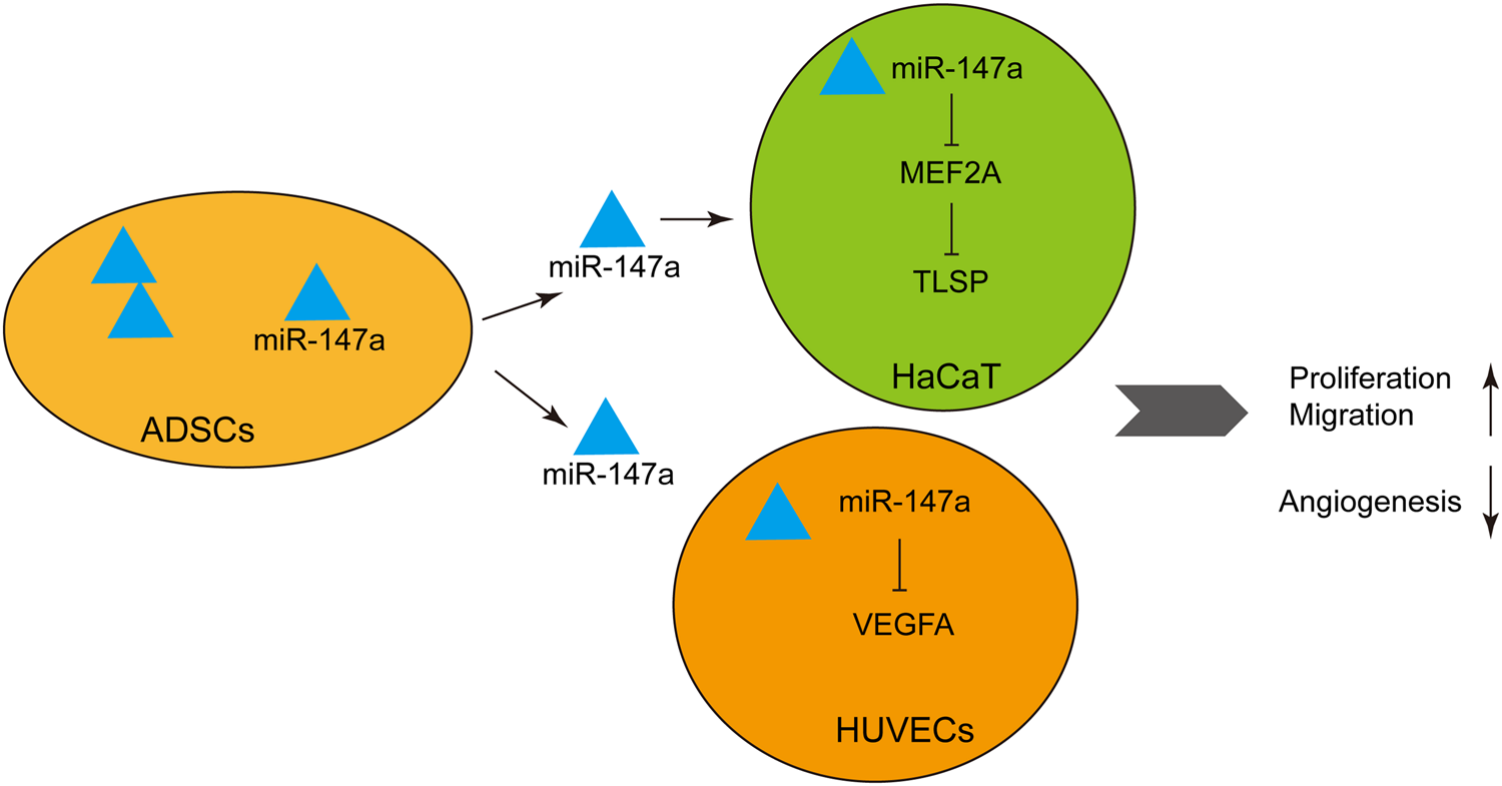
A schematic graph of exosomes derived from ADSCs-overexpressing miR-147a involved in the pathogenesis of AD.

In sum, our current study demonstrated that miR-147a-overexpressing ADSCs-derived exosomes repressed inflammatory injury and pathological angiogenesis by targeting VEGFA and MEF2A-TSLP axis to alleviate AD (Fig. 8). Certainly, there are some limitations to our study. We only studied the therapeutic effect of ADSCs-derived exosomes on AD and its related mechanisms at the preclinical level, without confirming the efficacy of ADSCs-derived exosomes at the clinical level. In future studies, we will study the therapeutic effect of miR-147a-overexpressing ADSCs-derived exosomes on AD at the clinical level, which makes our conclusions more credible.

## Supplementary Information

Supplementary Information.

## Data Availability

All data generated or analyzed during this study are included in this published article.

## Abbreviations

AD: Atopic dermatitis
VEGF: Vascular endothelial growth factor
MSCs: Mesenchymal stem cells
ADSCs: Adipose-derived stem cells
miRNAs: MicroRNAs
LPS: Lipopolysaccharide
MEF2A: Myocyte enhancer factor 2A
TSLP: Thymic stromal lymphopoietin
HUVECs: Human umbilical vein endothelial cells
ELISA: Enzyme-linked immunosorbent assay
TUNEL: TdT-mediated dUTP nick-end labeling
CCK-8: Cell counting kit-8
TEM: Transmission electron microscopy
ChIP: Chromatin immunoprecipitation
RT-qPCR: Real time quantitative polymerase chain reaction
SD: Standard deviation
ANOVA: Analysis of variance

## References

[1] Klonowska J, Gleń J, Nowicki RJ, Trzeciak M (2018). New cytokines in the pathogenesis of atopic dermatitis-new therapeutic targets. Int. J. Mol. Sci..

[2] Kim JE, Kim JS, Cho DH, Park HJ (2016). Molecular mechanisms of cutaneous inflammatory disorder: Atopic dermatitis. Int. J. Mol. Sci..

[3] Kapugi M, Cunningham K (2019). Corticosteroids. Orthop. Nurs..

[4] Nezamololama, N., Fieldhouse, K., Metzger, K., & Gooderham M. Emerging systemic JAK inhibitors in the treatment of atopic dermatitis: A review of abrocitinib, baricitinib, and upadacitinib. *Drugs Context* **9**10.7573/dic.2020-8-5 (2020). 10.7573/dic.2020-8-5 PMC7673622 33240390

[5] Blauvelt A, Teixeira HD, Simpson EL, Costanzo A, De Bruin-Weller M, Barbarot S (2021). Efficacy and Safety of upadacitinib vs dupilumab in adults with moderate-to-severe atopic dermatitis: A randomized clinical trial. JAMA Dermatol..

[6] Seok SH, An JH, Shin JU, Lee HJ, Kim DH, Yoon MS (2020). Facial redness in atopic dermatitis patients treated with dupilumab: A case series. Allergy, Asthma Immunol. Res..

[7] Paller AS, Kabashima K, Bieber T (2017). Therapeutic pipeline for atopic dermatitis: End of the drought?. J. Allergy Clin. Immunol..

[8] Ungar B, Pavel AB, Li R, Kimmel G, Nia J, Hashim P (2021). Phase 2 randomized, double-blind study of IL-17 targeting with secukinumab in atopic dermatitis. J. Allergy Clin. Immunol..

[9] Vandeghinste N, Klattig J, Jagerschmidt C, Lavazais S, Marsais F, Haas JD (2018). Neutralization of IL-17C reduces skin inflammation in mouse models of psoriasis and atopic dermatitis. J. Invest. Dermatol..

[10] Guttman-Yassky E, Krueger JG (2018). IL-17C: A unique epithelial cytokine with potential for targeting across the spectrum of atopic dermatitis and psoriasis. J. Invest. Dermatol..

[11] Genovese A, Detoraki A, Granata F, Galdiero MR, Spadaro G, Marone G (2012). Angiogenesis, lymphangiogenesis and atopic dermatitis. Chem. Immunol. Allergy.

[12] Lee HJ, Hong YJ, Kim M (2021). Angiogenesis in chronic inflammatory skin disorders. Int. J. Mol. Sci..

[13] Pelekanos RA, Li J, Gongora M, Chandrakanthan V, Scown J, Suhaimi N (2012). Comprehensive transcriptome and immunophenotype analysis of renal and cardiac MSC-like populations supports strong congruence with bone marrow MSC despite maintenance of distinct identities. Stem Cell Res..

[14] Minteer D, Marra KG, Rubin JP (2013). Adipose-derived mesenchymal stem cells: Biology and potential applications. Adv. Biochem. Eng. Biotechnol..

[15] Park HS, Son HY, Choi MH, Son Y, Kim S, Hong HS (2019). Adipose-derived stem cells attenuate atopic dermatitis-like skin lesions in NC/Nga mice. Exp. Dermatol..

[16] Lee HJ, Jung M, Kim JH, Yoon NY, Choi EH (2012). The effect of adipose-derived stem cell-cultured media on oxazolone treated atopic dermatitis-like murine model. Ann. Dermatol..

[17] Nakano M, Nagaishi K, Konari N, Saito Y, Chikenji T, Mizue Y (2016). Bone marrow-derived mesenchymal stem cells improve diabetes-induced cognitive impairment by exosome transfer into damaged neurons and astrocytes. Sci. Rep..

[18] Nie JY, Zhu YZ, Wang JW, Hu X, Wang ZH, Wu S (2021). Preparing adipogenic hydrogel with neo-mechanical isolated adipose-derived extracellular vesicles for adipose tissue engineering. Plast. Reconstr. Surg..

[19] Laloze J, Lupon E, Girard P, Gandolfi S, Fiévet L, Desmoulière A (2021). Supplementation with extracellular vesicles derived from adipose-derived stem cells increases fat graft survival and browning in mice: A cell-free approach to construct beige fat from white fat grafting. Plast. Reconstr. Surg..

[20] Doeppner TR, Herz J, Görgens A, Schlechter J, Ludwig AK, Radtke S (2015). Extracellular vesicles improve post-stroke neuroregeneration and prevent postischemic immunosuppression. Stem Cells Transl Med..

[21] Phinney DG, Pittenger MF (2017). Concise review: MSC-derived exosomes for cell-free therapy. Stem Cells.

[22] Masyuk AI, Masyuk TV, Larusso NF (2013). Exosomes in the pathogenesis, diagnostics and therapeutics of liver diseases. J. Hepatol..

[23] Lan T, Luo M, Wei X (2021). Mesenchymal stem/stromal cells in cancer therapy. J. Hematol. Oncol..

[24] Park KY, Han HS, Park JW, Kwon HH, Park GH, Seo SJ (2022). Exosomes derived from human adipose tissue-derived mesenchymal stem cells for the treatment of dupilumab-related facial redness in patients with atopic dermatitis: A report of two cases. J. Cosmet. Dermatol..

[25] Cho BS, Kim JO, Ha DH, Yi YW (2018). Exosomes derived from human adipose tissue-derived mesenchymal stem cells alleviate atopic dermatitis. Stem Cell Res. Ther..

[26] Specjalski K, Jassem E (2019). MicroRNAs: Potential biomarkers and targets of therapy in allergic diseases?. Arch. Immunol. Ther. Exp. (Warsz)..

[27] Rożalski M, Rudnicka L, Samochocki Z (2016). MiRNA in atopic dermatitis. Postepy Dermatol. Alergol..

[28] Liew WC, Sundaram GM, Quah S, Lum GG, Tan JSL, Ramalingam R (2020). Belinostat resolves skin barrier defects in atopic dermatitis by targeting the dysregulated miR-335:SOX6 axis. J. Allergy Clin. Immunol..

[29] Liu M, Li W, Song F, Zhang L, Sun X (2020). Silencing of lncRNA MIAT alleviates LPS-induced pneumonia via regulating miR-147a/NKAP/NF-κB axis. Aging (Albany NY).

[30] Li XQ, Huang TY (2021). Notoginsenoside R1 alleviates high glucose-induced inflammation and oxidative stress in HUVECs via upregulating miR-147a. Kaohsiung J. Med. Sci..

[31] Gröne A (2002). Keratinocytes and cytokines. Vet. Immunol. Immunopathol..

[32] Ständer S (2021). Atopic dermatitis. N. Engl. J. Med..

[33] Zhang B, Wei CY, Chang KK, Yu JJ, Zhou WJ, Yang HL (2017). TSLP promotes angiogenesis of human umbilical vein endothelial cells by strengthening the crosstalk between cervical cancer cells and eosinophils. Oncol Lett..

[34] Fu X, Liu G, Halim A, Ju Y, Luo Q, Song AG (2019). Mesenchymal stem cell migration and tissue repair. Cells.

[35] Lin YC, Grahovac T, Oh SJ, Ieraci M, Rubin JP, Marra KG (2013). Evaluation of a multi-layer adipose-derived stem cell sheet in a full-thickness wound healing model. Acta Biomater..

[36] Shin TH, Lee BC, Choi SW, Shin JH, Kang I, Lee JY (2017). Human adipose tissue-derived mesenchymal stem cells alleviate atopic dermatitis via regulation of B lymphocyte maturation. Oncotarget.

[37] Kim M, Lee SH, Kim Y, Kwon Y, Park Y, Lee HK (2018). Human adipose tissue-derived mesenchymal stem cells attenuate atopic dermatitis by regulating the expression of MIP-2, miR-122a-SOCS1 axis, and Th1/Th2 responses. Front. Pharmacol..

[38] Wu P, Zhang B, Shi H, Qian H, Xu W (2018). MSC-exosome: A novel cell-free therapy for cutaneous regeneration. Cytotherapy.

[39] Wei Z, Qiao S, Zhao J, Liu Y, Li Q, Wei Z (2019). miRNA-181a over-expression in mesenchymal stem cell-derived exosomes influenced inflammatory response after myocardial ischemia-reperfusion injury. Life Sci..

[40] Xu Y, Zhan X (2022). lncRNA KCNQ1OT1 regulated high glucose-induced proliferation, oxidative stress, extracellular matrix accumulation, and inflammation by miR-147a/SOX6 in diabetic nephropathy (DN). Endocr. J..

[41] Spinosa M, Lu G, Su G, Bontha SV, Gehrau R, Salmon MD (2018). Human mesenchymal stromal cell-derived extracellular vesicles attenuate aortic aneurysm formation and macrophage activation via microRNA-147. Faseb J..

[42] Brunner PM, Suárez-Fariñas M, He H, Malik K, Wen HC, Gonzalez J (2017). The atopic dermatitis blood signature is characterized by increases in inflammatory and cardiovascular risk proteins. Sci. Rep..

[43] Clausen ML, Kezic S, Olesen CM, Agner T (2020). Cytokine concentration across the stratum corneum in atopic dermatitis and healthy controls. Sci. Rep..

[44] Giacomin PR, Siracusa MC, Walsh KP, Grencis RK, Kubo M, Comeau MR (2012). Thymic stromal lymphopoietin-dependent basophils promote Th2 cytokine responses following intestinal helminth infection. J. Immunol..

[45] Berna R, Mitra N, Lou C, Wan J, Hoffstad O, Wubbenhorst B (2021). TSLP and IL-7R variants are associated with persistent atopic dermatitis. J. Invest. Dermatol..

[46] Soumelis V, Reche PA, Kanzler H, Yuan W, Edward G, Homey B (2002). Human epithelial cells trigger dendritic cell mediated allergic inflammation by producing TSLP. Nat. Immunol..

[47] Xiong Y, Wang L, Jiang W, Pang L, Liu W, Li A (2019). MEF2A alters the proliferation, inflammation-related gene expression profiles and its silencing induces cellular senescence in human coronary endothelial cells. BMC Mol Biol..

