# Supplementary figures and images for: Exosomes with overexpressed miR 147a suppress angiogenesis and infammatory injury in an experimental model of atopic dermatitis
Source: Sci Rep. 2023 Jun 1;13:8904. doi: 10.1038/s41598-023-34418-y (PMC10235063; doi:10.1038/s41598-023-34418-y)

FIG4D

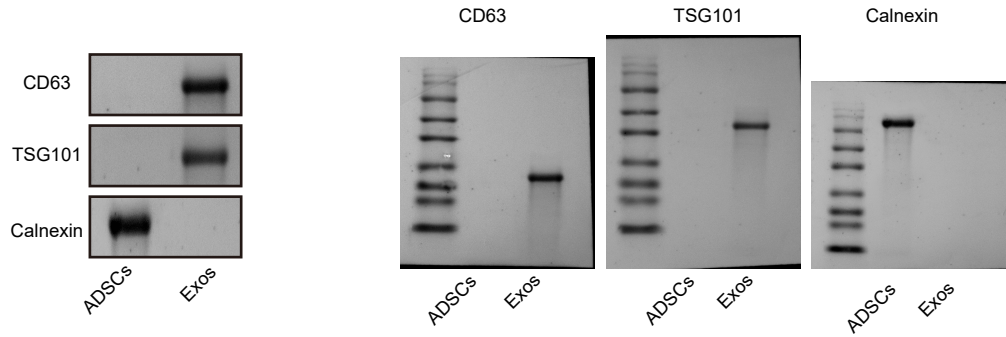

FIG7D

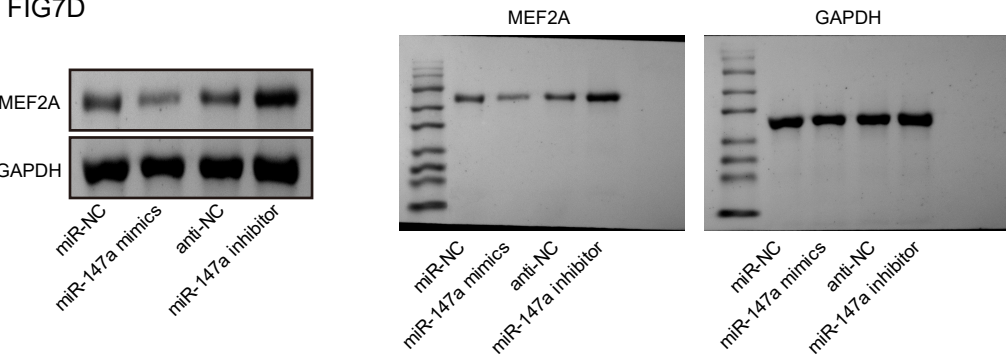

FIG7I

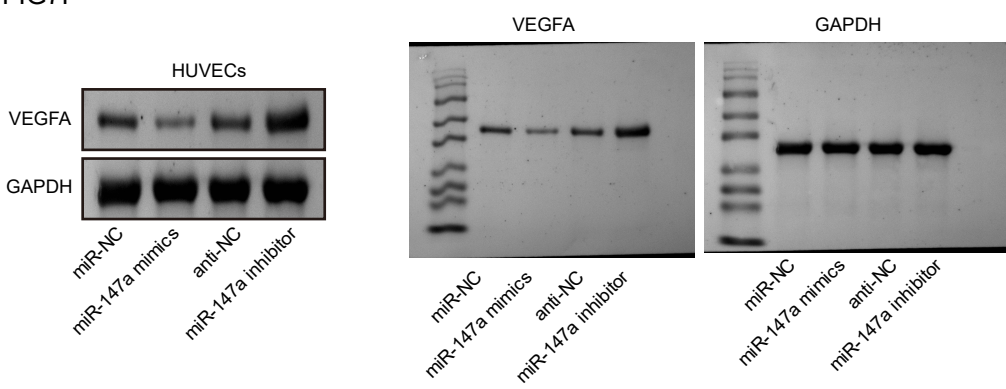

Supplement: Supplementary file 1 — Supplementary Information. [file 41598_2023_34418_MOESM1_ESM.pdf]
